# PLGA nanoparticles modified with a BBB-penetrating peptide co-delivering Aβ generation inhibitor and curcumin attenuate memory deficits and neuropathology in Alzheimer's disease mice

**DOI:** 10.18632/oncotarget.20944

**Published:** 2017-09-15

**Authors:** Na Huang, Shuai Lu, Xiao-Ge Liu, Jie Zhu, Yu-Jiong Wang, Rui-Tian Liu

**Affiliations:** ^1^ State Key Laboratory of Biochemical Engineering, Institute of Process Engineering, Chinese Academy of Sciences, Beijing 100190, China; ^2^ Key Laboratory of Ministry of Education for Protection and Utilization of Special Biological Resources in Western China, Ningxia University, Yinchuan 750021, China; ^3^ University of Chinese Academy of Sciences, Beijing 100049, China

**Keywords:** Alzheimer’s disease, nanoparticles, β-amyloid, curcumin, peptide

## Abstract

Alzheimer's disease (AD) is the most common form of dementia, characterized by the formation of extracellular senile plaques and neuronal loss caused by amyloid β (Aβ) aggregates in the brains of AD patients. Conventional strategies failed to treat AD in clinical trials, partly due to the poor solubility, low bioavailability and ineffectiveness of the tested drugs to cross the blood-brain barrier (BBB). Moreover, AD is a complex, multifactorial neurodegenerative disease; one-target strategies may be insufficient to prevent the processes of AD. Here, we designed novel kind of poly(lactide-co-glycolic acid) (PLGA) nanoparticles by loading with Aβ generation inhibitor S1 (PQVGHL peptide) and curcumin to target the detrimental factors in AD development and by conjugating with brain targeting peptide CRT (cyclic CRTIGPSVC peptide), an iron-mimic peptide that targets transferrin receptor (TfR), to improve BBB penetration. The average particle size of drug-loaded PLGA nanoparticles and CRT-conjugated PLGA nanoparticles were 128.6 nm and 139.8 nm, respectively. The results of Y-maze and new object recognition test demonstrated that our PLGA nanoparticles significantly improved the spatial memory and recognition in transgenic AD mice. Moreover, PLGA nanoparticles remarkably decreased the level of Aβ, reactive oxygen species (ROS), TNF-α and IL-6, and enhanced the activities of super oxide dismutase (SOD) and synapse numbers in the AD mouse brains. Compared with other PLGA nanoparticles, CRT peptide modified-PLGA nanoparticles co-delivering S1 and curcumin exhibited most beneficial effect on the treatment of AD mice, suggesting that conjugated CRT peptide, and encapsulated S1 and curcumin exerted their corresponding functions for the treatment.

## INTRODUCTION

Alzheimer's disease (AD) is characterized by ageing-associated extracellular accumulation of beta-amyloid (Aβ) aggregates, intracellular neurofibrillary tangles and progressive memory loss [1, 2]. Spontaneous self-aggregation of Aβ, a peptide derived from proteolysis of amyloid precursor protein (APP), plays a critical role in the etiology of AD [3]. The formed Aβ aggregates may trigger neurotoxicity by inducing inflammation responses and oxidative stress in the brains of AD patients, leading to memory loss and cognitive defect [4, 5]. Therefore, decreasing Aβ production and inhibiting inflammation and oxidative stress in brains are feasible therapeutic strategies for the treatment of AD.

Inhibiting the activity of β-amyloid precursor protein cleaving enzyme 1 (BACE1), a rate-limiting enzyme in the generation of Aβ from APP, has been proved to be an effective way to lower Aβ levels in AD [6, 7]. However, the inhibitors targeting BACE1 may induce serious side effects [8, 9], as BACE1 has some other substrates and exerts many physiological functions [10, 11]. For example, BACE1 is required for remyelination of regenerated axons of peripheral nerves and is also important for normal glomerulus formation in the olfactory bulb. [12, 13]. Our previously-reported peptide S1, binding to the cleavage site of β-secretase on APP rather than BACE1, may avoid the unexpected harmful effects [14]. Besides Aβ-production inhibitors, many neuroprotective compounds also showed promise in AD treatment [15–17]. Curcumin, a polyphenolic compound from the herbaceous plant *Curcuma longa*, showed various neuroprotective effects by decreasing neuroninflammation, oxidative stress and the levels of nitric oxide [18, 19]. However, several clinical trials of curcumin failed, possibly due to its poor water solubility and low brain bioavailability [20].

AD pathogenic process is associated with many factors, it might be more beneficial to treat AD using combined medicines targeting multiple factors [21, 22]. An ideal carrier may simultaneously delivery two or more drugs and increase water solubility, bioavailability and *in vivo* half-life of loaded drugs including peptides, compounds, proteins and antibodies, exerting more therapeutic effects on AD [23]. PLGA nanoparticle carrier, approved by FDA for the treatment of various diseases, is one of the most promising pharmaceutical delivery platforms due to the excellent biocompatibility, low cytotoxicity and biodegradability [24]. Consistently, several kinds of drug-loaded PLGA nanoparticles were reported to reverse cognitive deficits in AD transgenic mouse model [25–27]. However, PLGA-based nanotherapeutics still suffers from a low BBB penetration ability.

To improve the penetration of drugs across BBB, several approaches such as adsorptive-mediated transcytosis, receptor-mediated transport, cell-mediated endocytosis, and peptide vectors were developed [28]. Among these approaches, peptide vectors are suitable for modifying PLGA nanoparticles by chemical conjugation due to their low molecular weight [23]. The iron-mimic cyclic peptide, CRTIGPSVC (CRT), is able to target protein complex of transferrin and transferrin receptor (TfR) and improve BBB penetration of drugs as a safe and high efficient peptide vector [29, 30]. Here, we designed novel PLGA nanoparticles to simultaneously delivery S1 peptide, curcumin, which were conjugated with CRT to increase brain bioavailability, *in vivo* half-life and therapeutic effect of the encapsulated drugs.

## RESULTS

### Characterization of PLGA nanoparticles

**Figure 1:**
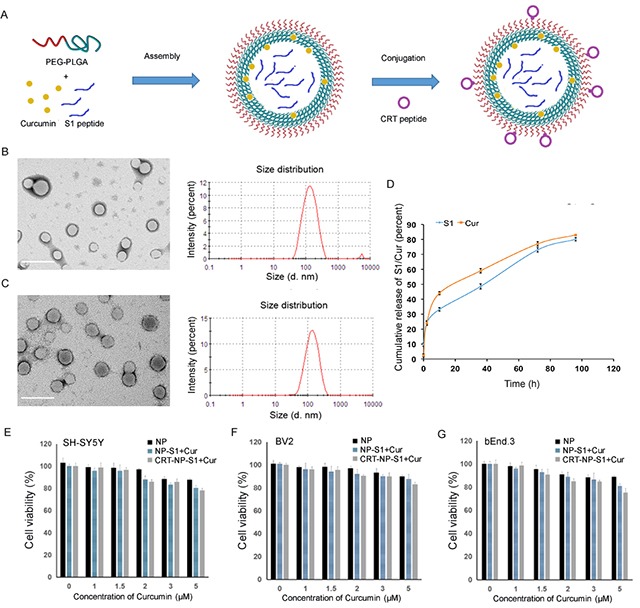
Characterization of PLGA nanoparticles **(A)** Schematic illustration of the PLGA NP fabrication. The PLGA NPs were prepared by self-assembly of PEG-PLGA with curcumin and S1 peptide, and then were conjugated with CRT peptide. The morphologies and diameters of NP-S1+Cur **(B)** and CRT-NP-S1+Cur **(C)** were determined by TEM and DLS, respectively (scale bar, 200 nm). The release profiles of curcumin and S1 from CRT-NP-S1+Cur in PBS (pH 7.4, containing 0.5% polysorbitol-80) at 37°C were detected by measuring absorbance at 430 nm and BCA, respectively **(D)**. The cytotoxicity of NP-S1+Cur conjugated with or without CRT was determined via MTT assay by adding NPs to SH-SY5Y cells **(E)**, BV2 cells **(F)** and bEnd.3 cells **(G)**. The data shown are expressed as the percentage of control values from three independent experiments with each experimental value being the average of six replicate wells.

S1- and curcumin-loaded PLGA nanoparticles conjugated with or without CRT were imaged by TEM. The results showed that PLGA coating provided a smooth surface to the nanoparticles and the synthesized nanoparticles with spherical shape were well dispersed with little adherence each other (Figure 1B and 1C). The DLS results demonstrated that the synthesized nanoparticles were monodispersed and the mean size was 128.6 ± 15.8 nm (Figure 1B). After conjugation with the CRT peptide, the mean size was increased to 139.8 ± 15.2 nm (Figure 1C). The zeta-potential of NP-S1+Cur and CRT-NP-S1+Cur was -32.4 mV and -25.7 mV, respectively (Table 1). BCA analysis showed that the conjugation efficiency of CRT peptide to nanoparticles was 35.3 ± 0.7%. The percentages of curcumin encapsulation efficiency of NP-S1+Cur and CRT-NP-S1+Cur were 23.2 ± 5.3% and 21.4 ± 6.9%, respectively (Table 1). The percentages of S1 peptide encapsulation efficiency of NP-S1+Cur and CRT-NP-S1+Cur were 14.7 ± 3.4% and 13.2 ± 3.6%, respectively (Table 1). The encapsulated curcumin and S1 was able to release from the PLGA NPs at a stable rate in PBS buffer (Figure 1D). The NPs were highly stable in 10 mM PBS buffer for three months without aggregation or significant change of particle size (Supplementary Figure 1).

**Table 1:** Characterization of nanoparticles

Nanoparticle	Average size	Zeta-potential	Curcumin EE%	S1 peptide EE%
NP-S1+Cur	128.6 nm	-32.4 mV	23.2 ± 5.3%	14.7± 3.4%
CRT-NP-S1+Cur	139.8 nm	-25.7 mV	21.4 ± 6.9%	13.2 ± 3.6%

To measure the cytotoxicity of the PLGA nanoparticles, MTT assay was performed by adding five concentrations of each PLGA nanoparticle (NP, NP-S1, NP-Cur, NP-S1+Cur and CRT-NP-S1+Cur) to neuroblastoma SH-SY5Y cells, microglial BV2 cells and brain microvascular bEnd.3 cells. Compared with cells alone, all the cells co-cultured with different kinds of NPs at five concentrations showed a viability of more than 75%, suggesting that our various PLGA nanoparticles did not possess significant cytotoxic effect on these cells (Figure 1E–1G).

### The permeation of the PLGA nanoparticles across the brain blood barrier *in vitro* and *in vivo*

**Figure 2:**
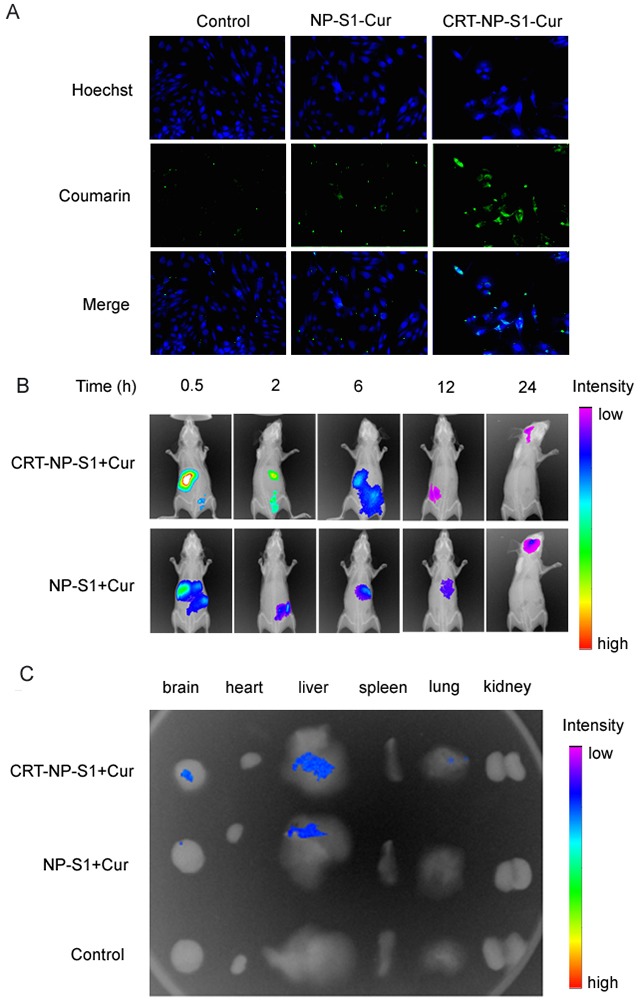
CRT peptide increased the uptake of PLGA NPs by bEnd 3 cells and improved the penetration of PLGA NP to brains. **(A)** Coumarin-6-labeled PLGA NP control, NP-S1+Cur and CRT-NP-S1+Cur were added to bEnd.3 cells. The fluorescence in cells was detected by a confocal microscope (scale bar, 20 μm). **(B)** ICG-labeled NP-S1+Cur and CRT-NP-S1+Cur were injected to nude mice via the tail vein. The fluorescence was detected by IVIS spectrum imaging system at different time points. **(C)** The mice were sacrificed and the fluorescence in various organs was detected.

To determine the permeation of the PLGA nanoparticles across the brain-blood barrier, we used a single layer of brain microvascular bEnd.3 cells as a BBB model to measure the uptake of coumarin-6-loaded PLGA nanoparticles into the cells. The results showed that few NP control and NP-S1+Cur nanoparticles were taken up into the cells and distributed inside the cells, while a greater number of CRT-NP-S1+Cur nanoparticles with high fluorescence intensity were observed in the cells, suggesting that the CRT peptide increased the permeation of PLGA NPs across BBB model (Figure 2A, Supplementary Figure 2). To study the biodistribution of PLGA nanoparticle in *vivo*, we intravenously administrated ICG-labeled NP and CRT-NP via tail vein and measured the fluorescence intensity using an *in vivo* bioluminescence imaging system. The biodistribution of both kinds of nanoparticles time-dependently changed, and more CRT-NPs accumulated in the mouse brain comparing with PLGANPs without CRT (Figure 2B). Consistently, more CRT-NP-S1+Cur were observed in the brains of the mice sacrificed at 24 h after administration of PLGA NPs, further indicating that CRT increased the penetration of PLGA NPs to mouse brains (Figure 2C).

### NPs attenuates cognitive impairments in transgenic AD mice

**Figure 3:**
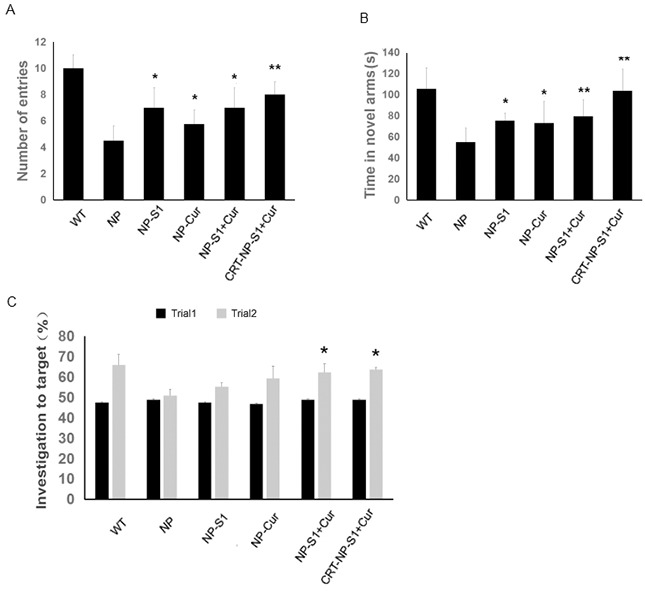
PLGA NPs attenuated memory defects in AD model mice The eight-month-old transgenic AD mice were treated with NP control, NP-S1, NP-Cur, NP-S1+Cur and CRT-NP-S1+Cur, respectively, and their memory was determined via Y maze **(A, B)** and NOR **(C)**. The number of entries to the novel arm by the mice (A) and the time in the novel arm spent by the mice (B) were measured. (C) The investigation to novel target by mice was determined via NOR test. (*, P < 0.05, **, P < 0.01, compared with NP control-treated AD mice).

Y-maze and new object recognition test were used to investigate the effects of NPs on learning and memory ability in AD mice. Compared with wild type mice, the AD mice treated with NP control showed remarkably less time in novel arm and low number of entries to the novel arm. However, NP-S1, NP-Cur, NP-S1+Cur and CRT-NP-S1+Cur significantly increased the time in novel arm and the numbers of entries to novel arm while CRT-NP-S1+Cur exhibited the most beneficial effects (Figure 3A and 3B). Consistently, the AD mice treated with NP control did not show obvious improvement in investigation to novel target in NOR test. But the treatment with NP-S1-Cur and CRT-NP-S1+Cur improved the investigation of AD mice to novel target (Figure 3C). These results indicated that S1 and curcumin-loaded PLGA NPs passed the BBB and attenuated memory deficits in AD mice, and CRT increased the BBB penetration of NPs and improved the beneficial effects of S1 peptide and curcumin on AD mice.

### PLGA NPs decreased Aβ burden in the brains of AD mice

**Figure 4:**
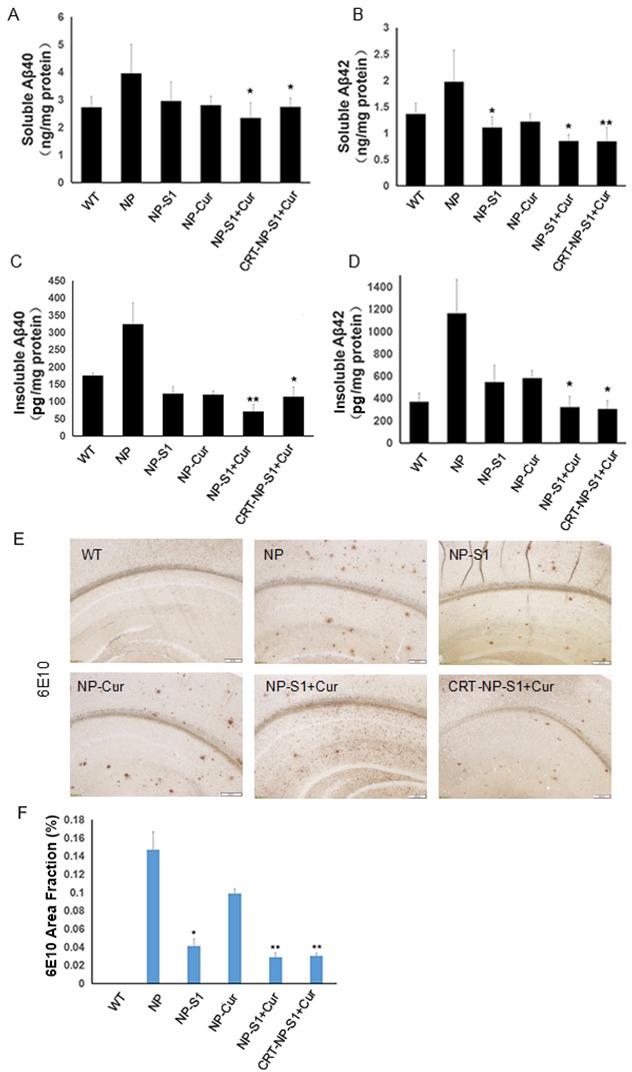
PLGA NPs reduced Aβ40 and Aβ42 levels in AD mouse brains The levels of soluble Aβ40 **(A)** and Aβ42 **(B)**, insoluble Aβ40 **(C)** and Aβ42 **(D)** in the brain lysates of AD mice treated with NP control, NP-S1, NP-Cur, NP-S1+Cur and CRT-NP-S1+Cur were detected by Aβ40 and Aβ42 sandwich ELISA kits, respectively. The senile plaques in the brains of AD transgenic mice treated with NP control, NP-S1, NP-Cur, NP-S1+Cur and CRT-NP-S1+Cur were detected by immunohistochemistry **(E)** and quantitatively analyzed by Image-Pro Plus software **(F)** (*, P < 0.05, **, P < 0.01, compared with NP control-treated AD mice).

The levels of soluble and insoluble Aβ40 and Aβ42 in AD mouse brains were detected by ELISA assay kits. The levels of soluble and insoluble of Aβ40 and Aβ42 in the PLGA NP control-treated AD mice were remarkably higher than that in wild type mice. However, compared with PLGA NP control, NP-S1, NP-Cur, NP-S1+Cur and CRT-NP-S1+Cur significantly decreased both soluble and insoluble Aβ levels (Figure 4A–4D). Consistent with the above results, the immunohistochemistry results showed that PLGA NPs with encapsulated S1 significantly decreased the Aβ deposit burden in the AD mouse brains (Figure 4E and 4F), implying that the S1 peptide delivered by PLGA nanoparticle effectively inhibited Aβ production in *vivo*. Consistent with this result, NPs loaded with S1 peptide were able to inhibit APP cleavage *in vitro* (Supplementary Figure 3).

### PLGA NPs suppressed microgliosis, astrogliosis and increased synapse number in the brains of AD transgenic mice

**Figure 5:**
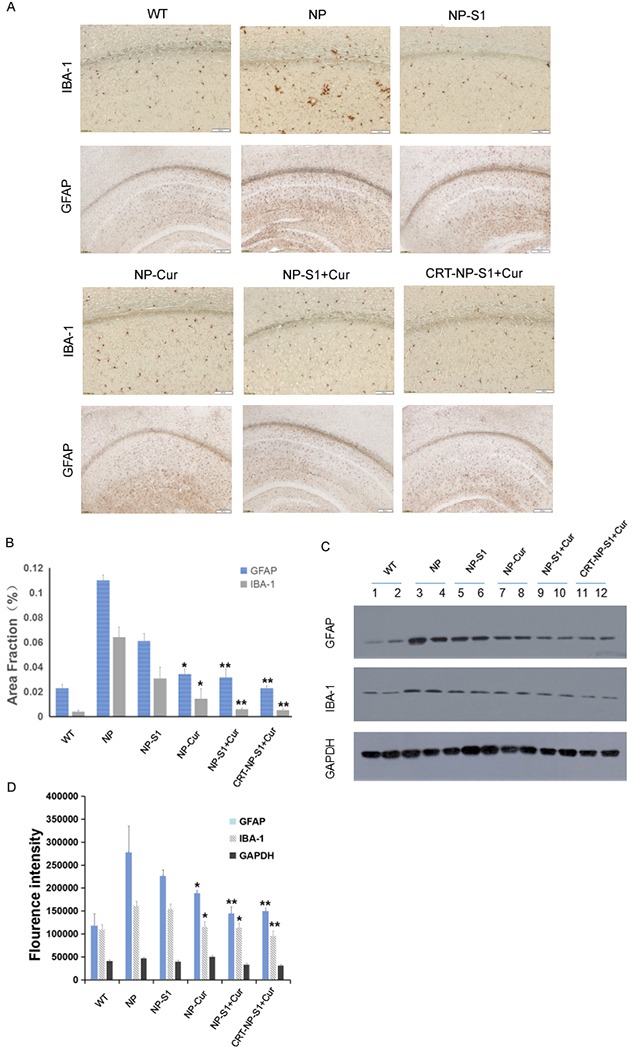
PLGA NPs reduced microgliosis and astrogliosis in AD mice **(A)** Microgliosis and astrogliosis in the brains of AD mice treated with NP control, NP-S1, NP-Cur, NP-S1+Cur and CRT-NP-S1+Cur were detected by IBA-1 and GFAP immunostaining, and qualified by Image-Pro Plus software **(B)**, respectively. The protein levels of GFAP and Iba-1 in the brain lysates of AD transgenic mice were detected by western blot **(C)** and quantitatively analyzed by ImageQuant software **(D)**. GAPDH was used as a loading control (*, P < 0.05, **, P < 0.01, ***, P < 0.001, compared with AD control mice).

**Figure 6:**
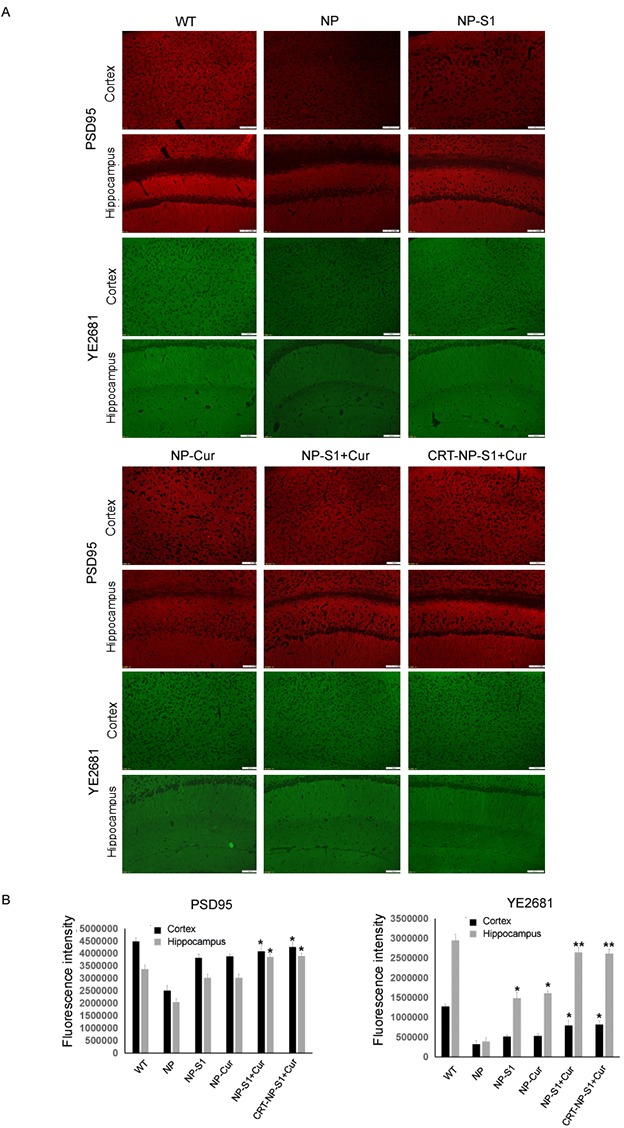
PLGA NPs increased synapse number in AD mouse brains The AD mice were treated with NP control, NP-S1, NP-Cur, NP-S1+Cur and CRT-NP-S1+Cur, and the synapses in the cerebral cortex and hippocampus were detected by anti-PSD95 and YE2681 antibodies, respectively **(A)**. **(B)** The fluorescence intensity in (A) was quantitatively analyzed by Image-Pro Plus software (*, P < 0.05, **, P < 0.01, compared with NP control-treated AD mice).

Microgliosis and astrogliosis contributed to the pathogenic process of AD. Compared with wild type mice, more Iba-1-positive and GFAP-positive neuroglia were observed in the brain of AD mice treated with PLGA NP control. However, and number of active glia significantly decreased by the treatment of NP-S1, NP-Cur, NP-S1+Cur and CRT-NP-S1+Cur nanoparticles (Figure 5A and 5B). To further confirm these results, we detected the Iba-1 and GFAP protein levels in the mouse brains by western blot using anti-Iba-1 and anti-GFAP antibody. Consistent with the immunohistochemistry results, the treatment of NP, NP-Cur, NP-S1+Cur and CRT-NP-S1+Cur nanoparticles significantly decreased Iba-1 and GFAP protein levels (Figure 5C and 5D), indicating that S1 and curcumin suppressed Aβ-induced activation of neuroglia *in vivo*. Synapse loss is another character in the brains of AD patient or mice. When treated with NP-S1, NP-Cur, NP-S1+Cur and CRT-NP-S1+Cur nanoparticles, the levels of synaptophysin detected by YE2681 and PSD95 were remarkably increased (Figure 6).

### PLGA NPs decreased cytokine production and oxidative stress *in vivo*

**Figure 7:**
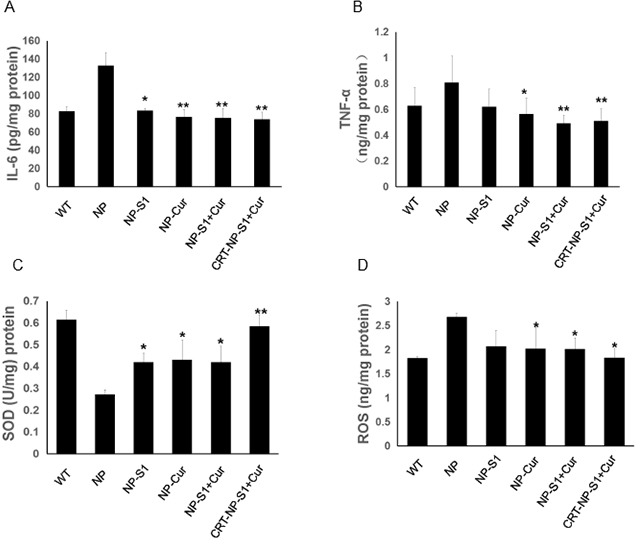
PLGA NPs decreased inflammatory cytokine production and attenuated oxidative stress in AD mice Levels of IL-6 **(A)** and TNF-α **(B)** in the brain lysates of AD mice treated with NP control, NP-S1, NP-Cur, NP-S1+Cur and CRT-NP-S1+Cur were determined using corresponding ELISA kits, respectively. The levels of SOD **(C)** and ROS **(D)** in the brain lysates of AD mice were determined using corresponding commercial kits, respectively (*, p < 0.05, **, p < 0.01, compared with NP control-treated AD mice).

The levels of cytokines in the mouse brains were detected by the ELISA assay kit. The levels of IL-6 and TNF-α in AD mice treated with PLGA nanoparticle control were increased comparing to wild type mice, whereas NP-S1, NP-Cur, NP-S1-Cur and CRT-NP-S1-Cur significantly decreased IL-6 and TNF-α levels (Figure 7A and 7B). To further investigate the effect of PLGA nanoparticles on oxidative stress in the brains of AD mice, the levels of SOD and ROS were measured. Compared to NP control, PLGA-drug nanoparticles significantly decreased the ROS level and increased the SOD level in the mouse brains (Figure 7C and 7D). These results indicated that S1 and curcumin reduced the cytokine production, restored antioxidant activity and reduced oxidative stress in AD mice, combination of S1 and curcumin improved the therapeutic effects and the CRT peptide further strengthened the treatment outcome.

## DISCUSSION

The size and encapsulation efficiency are important factors for the drug-loaded nanoparticles to exert function [31]. In this study, we applied emulsion-solvent evaporation method to prepare S1- and Curcumin-loaded PLGA NPs. Compared with other techniques of PLGA nanoparticle production, such as nanoprecipitation, spray drying, salting-out, and supercritical antisolvent precipitation, the emulsion-solvent evaporation method is most used in pharmaceutical industries to get controlled release formulations [32], and high entrapment of hydrophobic agents, such as curcumin. Accordingly, PLGA NPs overcame the limitation of low brain bioavailability of curcumin and sustained release of curcumin. However, the conventional emulsion-solvent evaporation method is unfavorable to encapsulate hydrophilic agents, such as S1. To simultaneously load both curcumin and S1, we employed double emulsion solvent evaporation method (w/o/w) with sonication [33]. The prepared PLGA NPs were in spherical shape and exhibited negative zeta potential. The diameter of these NPs was around 130 nm, which was acceptable for BBB penetration. Moreover, release of curcumin and S1 peptide from the PLGA NPs was at a stable rate. These results suggested that the double emulsion solvent evaporation method was feasible to prepare stable NPs to simultaneously load hydrophilic and hydrophobic agents.

BBB is an impediment for the delivery of therapeutic agents to brain. Although previous reports showed that small-size PLGA NPs crossed BBB, the efficiency of drug delivery to brain was still very low [34]. Some studies used anti-transferrin receptor antibody, cell-penetrating TAT peptide, or apolipoprotein peptide analog to modify NPs for brain targeting [35, 36]. However, these studies were mainly verified by *in vitro* cellular models and lacked more detailed *in vivo* investigations. In this study, we used an iron-mimic transferrin/transferrin receptor-targeting peptide CRT to conjugate with PLGA NPs. Consistent with the previous report [27, 37], the NP-S1+Cur crossed BBB with low efficiency; however, the CRT-NP-S1+Cur NPs showed remarkably-enhanced cellular uptake and BBB penetration ability *in vitro*. When administrated to mice, CRT-NP-S1+Cur showed stronger fluorescence in the mouse brains at 24 h after injection, further confirming that CRT conjugation to PLGA NPs markedly increased their BBB penetration. Additionally, comparing to previously-reported nanoparticles, our CRT-conjugated PLGA NPs exhibited less cytotoxicity to SH-SY5Y, BV-2 and bEnd.3 cells, suggesting that these NPs had good biocompatibility.

Reducing Aβ production is a promising strategy for AD treatment. We previously reported an APP adapter S1, which specifically bound to β-site on APP, significantly reduced APP cleavage and decreased Aβ generation *in vitro* and *in vivo* [14]. However, S1 is a short peptide with low half-life and low brain penetration. To surmount these obstacles of S1, we successfully encapsulated S1 into PLGA NPs, protecting S1 from hydrolysis and delivering S1 to brains. Activated microglial cells release several pro-inflammatory cytokines including TNF-α and IL-6, potentially contributing to neuronal cell dysfunction and apoptosis [38–40]. Curcumin is an antioxidant agent and showed promising therapeutic effect for AD by inhibiting inflammation and inducing adult neurogenesis via activation of Wnt/β-catenin signaling pathway [27, 41]. Besides its antioxidant effect, curcumin was reported to inhibit BACE1 expression and reduce Aβ production [42]. Consistent with this observation, curcumin loaded PLGA NPs also reduced the levels of Aβ40 and Aβ42 in mouse brains (Figure 6A–6D). Several studies showed that highly lipophilic curcumin nanoparticles improved brain bioavailability of curcumin and enhanced its therapeutic effect [27, 43]. To obtain better therapeutic effect for AD, we encapsulated curcumin together with peptide S1. Our animal test results demonstrated that the NPs loaded with S1 and curcumin significantly decreased Aβ burden, gliosis and inflammation in the brains of AD mice and increased spacial memory and recognition.

Multifunctional nanocarriers to regulate multiple pathological dysfunctions are promising for treating complex diseases. Previous studies revealed that nanoparticles loaded with several agents targeting multiple pathways gained synergistical effect on AD treatment [44]. A recent report showed that co-delivery of BACE1 antisense shRNA and a tau aggregation inhibiting peptide rescued memory loss of AD mice [45]. Although these nanoparticles remarkably relieved amyloid burden, there was no evidence that the oxidative stress and proinflammation in the AD mouse brain was reduced [45]. Herein, for the first time, we tried to co-delivery of Aβ production inhibition peptide and anti-inflammation compound to treat AD. The results showed that the CRT-conjugated NPs co-delivered S1 and curcumin displayed most beneficial effects, suggesting that CRT increased the BBB permeability of PLGA NPs, S1 and curcumin played a corresponding role in the treatment of AD mice by reducing Aβ generation, decreasing gliosis, proinflammation and cytokine production.

## MATERIALS AND METHODS

### Materials

L-S1 peptide (Cys &Cys bridge) and Cyclic peptide CRT (CRTIGPSVC) was synthesized by Chinese Peptides (Hangzhou, China). PLGA 20000-PEG 3400-NHS and PLGA (MW 23500) (for preparation of PLGA NP control) was purchased from Xi’an Ruixi Biological Technology (Xi’an, China). NHS (N-hydroxysuccinimide), EDC (N-(3-dimethylaminopropyl)-N’-ethylcarbodiimide hydrochloride) and N,N-diisopropylethylamine were purchased from Aladdin Reagent (Shanghai, China). Curcumin, coumarin-6, indocyanine green (ICG) and Hoechst 33258 were purchased from Sigma Aldrich (St. Louis, MO, USA). Male BALB/c nude mice weighing 25 ± 2 g and wild-type mice were obtained from HFK Bioscience (Beijing, China). Anti-Aβ antibody 6E10 (monoclonal against Aβ N-terminal) and 4G8 (monoclonal against Aβ17-24) were purchased from Signet Laboratories (Covance, Dedham, MA, USA). Anti-Iba-1 and Anti-GFAP antibodies were purchased from GeneTex (Alton Pkwylrvine, CA, USA). TNF-α and IL-1β ELISA kits were obtained from R&D Systems (Minneapolis, MN, USA). The assay kits for ROS and SOD were purchased from Beyotime Company (Jiangsu, China). The Aβ40 and Aβ42 measurement kits were purchased from IBL (Gunma, Japan).

### Preparation of PLGA nanoparticles

Emulsion–solvent evaporation method was used to prepare PLGA nanoparticles. Briefly, 100 μg of curcumin was added to the PLGA-PEG copolymer solution in dichloromethane and kept for 30 min at room temperature with intermittent vortexing. The mixture was sonicated at 40% amplitude for 45 seconds for emulsion (w/o), and then 2 mL of 1% sodium cholate was added and sonicated at 40% amplitude for one minute to obtain the second emulsion (w/o/w). The PLGA-curcumin mixture was added drop by drop to 0.5% sodium cholate aqueous solution which was used as a surfactant under moderate magnetic stirring for 4 h at 4°C, the NPs were collected by centrifugation at 14500 rpm for 45 min and washed three times with 0.01 M HEPES buffer (pH 7.0). The same procedure was used to prepare S1-loaded nanoparticles (NP-S1), S1- and curcumin-loaded nanoparticles (NP-S1+Cur), coumarin-6-labeled (NP-C6) and ICG-labeled nanoparticles (NP-ICG), respectively. For encapsulating hydrophilic solute, such as S1, 1% sodium cholate was used in the preparation of w/o emulsion. In case of encapsulating both hydrophobic and hydrophilic solute, curcumin and S1 were added at the same time in the w/o emulsion.

EDC-NHS coupling was used to conjugate the CRT peptide to the nanoparticles (CRT-NP-S1+Cur) for brain targeting. Firstly, 1 mL of S1 and curcumin-loaded nanoparticles (NP-S1+Cur) was mixed with 0.4 mmol NHS and 0.4 mmol EDC. The mixture was gently shaken for 1 h at room temperature. 50 μL of CRT peptide (1 mg/ml) was then added, and shaked for another 4 h. Finally, the CRT-conjugated nanoparticles with encapsulated S1 and curcumin (CRT-NP-S1+Cur) were collected by centrifugation at 14500 rpm for 45 min and washed three times with 0.01 M HEPES buffer (pH 7.0) to remove unreacted EDC.

### Nanoparticle characterization

The size distribution and zeta potential of different kinds of PLGA nanoparticles were determined by dynamic light scattering (DLS) (Zetasizer Nano ZS, Malvern, UK). Transmission electron microscopy (TEM) (HITACHI, HT7700, Japan) was used for the morphological examination of NPs by negative staining with sodium phosphotungstate solution, the imaging were observed at an acceleration voltage of 80 kV. To verify the conjugation of CRT peptide on the surface of NP, the NP samples were lyophilized via a freeze dryer with sorbitol addition at the concentration of 10% (w/v) as a cryoprotectant and then subjected to HPLC system to monitor the conjugation efficiency of CRT peptide to NPs.

Drug encapsulation efficiency (EE%) of S1 were determined by BCA protein assay. Briefly, 20 μL of samples (NP-S1+Cur and CRT-NP-S1+Cur) were firstly broken by sonication and the supernatant was collected after centrifugation at 13000 rpm for 45 min. 20 μL of the supernatant and 160 μL of BCA protein assay reagent were mixed well in a 96-well plate, and then incubated for 1 h at 37°C. Thereafter, the absorbance at 562 nm was measured via MD-M5 reader (MD, USA). Drug encapsulation efficiency (EE%) of curcumin were determined by measuring the absorbance at 430 nm according to the standard calibration curve plotted using standard concentrations of curcumin dissolved in ethanol.

To measure the release of curcumin and S1, 100 μL of NPs samples loaded with curcumin and S1 in 10mM HEPES buffer were centrifuged at 5600 rpm for 5 minutes after 0, 2, 12, 36, 72, 96 h, and the precipitation was resuspended in 200 μL methanol. Then, the solution was bath sonicated for 5 min to obtain the remaining S1 in the nanoparticles and centrifuged at 5600 rpm for 5 minutes. The supernatant was applied to mix with the BCA protein assay reagent in a 96-well plate, and then incubated for 1 h at 37°C. Thereafter, the absorbance at 562 nm was measured via MD-M5 reader to measure the level of S1. The release amount of S1 was calculated by total encapsulated amount of S1 subtracted by remaining amount of S1 in the NPs. The released curcumin in HEPES buffer were determined by measuring the absorbance at 430 nm according to the standard calibration curve plotted using standard concentrations of curcumin dissolved in ethanol.

### Determination of cytotoxicity of PLGA NPs

Cytotoxicity profile of the PLGA nanoparticles was detected via MTT assay using human neuroblastoma SH-SY5Y cells, mouse microglial BV2 cells and mouse brain capillary endothelial bEnd.3 cells, respectively. Three cell lines were maintained in Dulbecco's modified Eagle's medium (DMEM, Hyclone) with 10% fetal bovine serum (FBS) and 1% penicillin/streptomycin at 37°C under a 5% CO_2_ atmosphere, respectively. The cells were seeded in 96-well plates with approximately 10^4^ cells per 100 μL of medium per well. The plates were incubated at 37°C for 24 h to allow the cells to attach. The samples at 5 different concentrations were added to individual wells. The plates were incubated for another 48 h at 37°C. Cell viability was detected by adding 10 μL of 5 mg/ml MTT to each wells, and followed by the addition of 100 μL of crystal dissolvent. The absorbance at 560 nm was measured using MD-M5 microplate reader (MD, USA).

### Transport across *in vitro* BBB model

The bEnd.3 mouse brain endothelial cells were seeded onto glass cover slips of the 24-well plate at a density of 1 × 10^5^ cells per well, and then removed to a new 24-well plate after 24 h incubation. 400 μL of 200 ng/mL coumarin-6-labeled PLGA-NP, NP-S1+Cur, and CRT-NP-S1+Cur were added to the cell culture, respectively. Cells in DMEM without any addition of nanoparticles or dye were used as a control. After incubation, the cells were washed twice with PBS, fixed with paraformaldehyde (4.0% w/v) for 10 min at 4°C, permeabilized for 20 min with Triton X-100 (0.1% v/v), counter-stained with Hoechst 33342, and finally visualized with a confocal microscope (Zeiss Axio Imager Z2).

### *In vivo* real-time imaging

Male BALB/c nude mice were intravenously administrated with ICG-labeled NP-S1+Cur and CRT-NP-S1+Cur via the tail vein (ICG dose of 0.5 mg/kg), respectively. The fluorescent images were taken by the IVIS spectrum imaging system (Kodak In-Vivo Imaging System FX Pro, USA) at 0.5, 2, 6, 12, and 24 h post administration, and then the mice were sacrificed. The brains, hearts, livers, spleens, lungs and kidneys were washed with saline, and visualized under the *In Vivo* IVIS spectrum imaging system.

### Treatment of transgenic AD mice

Eight-month-old male AD model (APP/PS1dE9) mice (n = 5 per group) were treated with PLGA NPs at a PLGA dose of 2 mg/kg via intraperitoneal injection every two days for 3 weeks, and the age-matched wide type mice administrated with the same volume of PBS were used as a control. All animal tests were carried out in accordance with the China public health service guide for care and use of laboratory animals. Experiments involving mice and protocols were approved by the institutional animal care and use committee of Chinese academy of Sciences.

### Y-maze test

Y-maze testing consisted of 2 trials separated by an interval of 1 h. The first trial was 10 min in duration and allowed the mouse to explore only 2 arms (the start and familiar arms) of the maze, with the third arm (novel arm) blocked. The second trial was conducted after 1 h by putting the mice in the same starting arm as in trial 1 with free access to all 3 arms for 5 min. The total number of novel arm entries and time (in seconds) spent in the novel arm were obtained using a ceiling-mounted camera.

### New object recognition (NOR) test

NOR test is based on the spontaneous tendency of animals to exhibit more interactions with a novel rather than a familiar object. In the habituation phase, the animals were first familiarized with the open field arena in the absence of objects. During the familiarization period, each mouse was placed in the upper two corners of a box (50 cm × 50 cm × 25 cm) containing the same two object for 5 min, and returned quickly to its housing cage. Twenty-four hours later, mice are reintroduced into the arena containing two different objects, one of which was presented previously (familiar) and another was completely new one (novel). The time spent exploring and sniffing each object was recorded. Retention score is expressed as discrimination index (percentage of time exploring the novel object to the total time of object exploration). Animals without memory impairment spend a longer time investigating the novel object, giving a higher discrimination index.

### Immunohistochemical analysis

For immunohistochemistry, the paraffin-embedded sections were blocked with 10% normal goat serum and treated with 0.3% Triton X-100 in PBS to prevent nonspecific protein binding and penetrate cell membranes, and subsequently incubated with 6E10 antibody (1:100), anti-Iba-1 antibody (1:200), anti-GFAP antibody (1:100), anti-YE2681 antibody (1:100), and anti-PSD95 antibody (1:100) at 4°C overnight, respectively, and followed by incubation with HRP-labeled secondary antibodies at 37°C for 1 h. The targets were visualized with DAB and counterstained with hematoxylin. All images were acquired by an Olympus IX73 inverted microscope with DP80 camera (Olympus Corp., Shinjuku, Tokyo).

### Measurement of Aβ40/42

The brain extracts were obtained using a two-step extraction protocol according to a previously described method. In brief, one hemisphere (excluding the cerebellum) was homogenized in lysis buffer (50mM Tris-HCl, pH 7.6, 150 mM NaCl, 2 mM EDTA) with a cocktail of protease inhibitors. The homogenate was suspended in 2% SDS containing protease inhibitors and centrifuged at 4°C, 12,000 rpm for 1 h, and the supernatants were collected (soluble Aβ fraction). Afterward, the pellets were suspended in guanidine buffer (5.0 M guanidine-HCl/50 mM, Tris-HCl at pH 8.0) and centrifuged at 12,000 rpm for 1 h at 4°C; the supernatants were collected as insoluble Aβ fraction. Both soluble and insoluble (guanidine soluble) levels were quantified using Aβ40 and Aβ42 immunoassay kits according to the manufacturer's instructions and then standardized to the brain tissue weigh.

### Western blot

The protein samples were separated by 15% SDS-PAGE and transferred onto a nitrocellulose membrane (Millipore, USA). The membrane was then blocked with 5% nonfat dry milk in PBST and incubated with primary antibody anti-GFAP (1:1000), anti-Iba-1 (1:1000), and anti-GAPDH (1:1000), respectively. The blots were washed three times in TBST before incubation with the appropriate secondary antibody and then visualized by ECL chemiluminescene Kit (Pierce, Rockford, USA). The grey intensity of the western-blot bands was quantitatively analyzed by ImageQuant software.

### Measurement of TNF-α, IL-6 and SOD

The TNF-α and IL-6 levels in the cerebral homogenate were determined using the mouse TNF-α and IL-6 ELISA kits (NeoBioscience, Beijing, China) according to the manufacturer's protocols. Briefly, the brain extracts were added to a 96-well ELISA plate and then reacted with relevant primary antibodies and HRP-conjugated secondary antibodies. 3,3′,5,5′-Tetramethylbenzidine (TMB) was used as the substrate, and the absorbance was measured at 450 nm using MD-M5 microplate reader (Molecular Dynamics). The activity of SOD was detected by a total SOD assay kit according to the manufacturer's protocol. Briefly, brain extracts, nitroblue tetrazolium and enzyme-working solutions were prepared and added into a 96-well plate. The mixtures were incubated at 37°C for 20 min, and then the absorbance was assayed at 560 nm using an MD-M5 microplate reader.

### Statistical analysis

All the data were obtained from at least three separate experiments for each experimental condition and presented as mean ± standard deviation, and their statistical significance was analyzed by Student's t test or one-way ANOVA followed by Tukey's multiple comparison test.

## SUPPLEMENTARY MATERIALS FIGURES



## Abbreviations

(AD): Alzheimer's disease
(Aβ): amyloid β
(BBB): blood-brain barrier
(PLGA): poly(lactide-co-glycolic acid)
(ROS): reactive oxygen species
(SOD): super oxide dismutase
(APP): amyloid precursor protein
(BACE1): β-amyloid precursor protein cleaving enzyme 1
(TfR): transferrin receptor
(TEM): transmission electron microscopy
(EE%): encapsulation efficiency
(TMB): 3,3′,5,5′-Tetramethylbenzidine
