# Supplementary material for: PLGA nanoparticles modified with a BBB-penetrating peptide co-delivering Aβ generation inhibitor and curcumin attenuate memory deficits and neuropathology in Alzheimer's disease mice
Source: Oncotarget. 2017 Sep 15;8(46):81001–13. doi: 10.18632/oncotarget.20944 (PMC5655257; doi:10.18632/oncotarget.20944)
Supplement: Supplementary file 1 [file oncotarget-08-81001-s001.pdf]

PLGA nanoparticles modified with a BBB-penetrating peptide co-delivering Aβ generation inhibitor and curcumin attenuate memory deficits and neuropathology in Alzheimer's disease mice

SUPPLEMENTARY MATERIALS

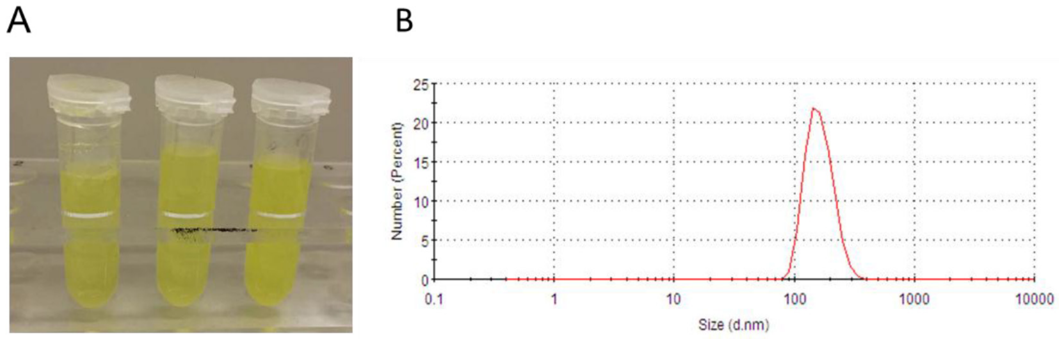

Supplementary Figure 1: Stability of PLGA nanoparticles. CRT-NP-S1-Cur NPs were kept in PBS buffer at 4 °C for 3 weeks and then the aggregation **(A)** and particle sizes **(B)** were measured.

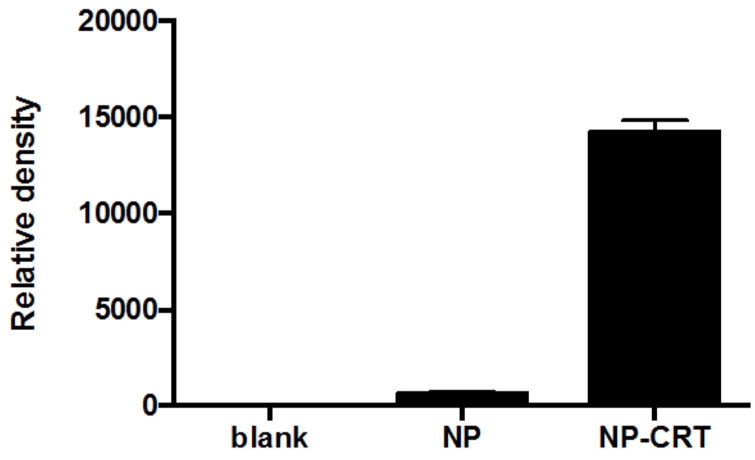

Supplementary Figure 2: Quantification of the fluorescence levels of coumarin-6-labeled PLGA NP control, NP-S1+Cur and CRT-NP-S1+Cur in bEnd. 3 cells.

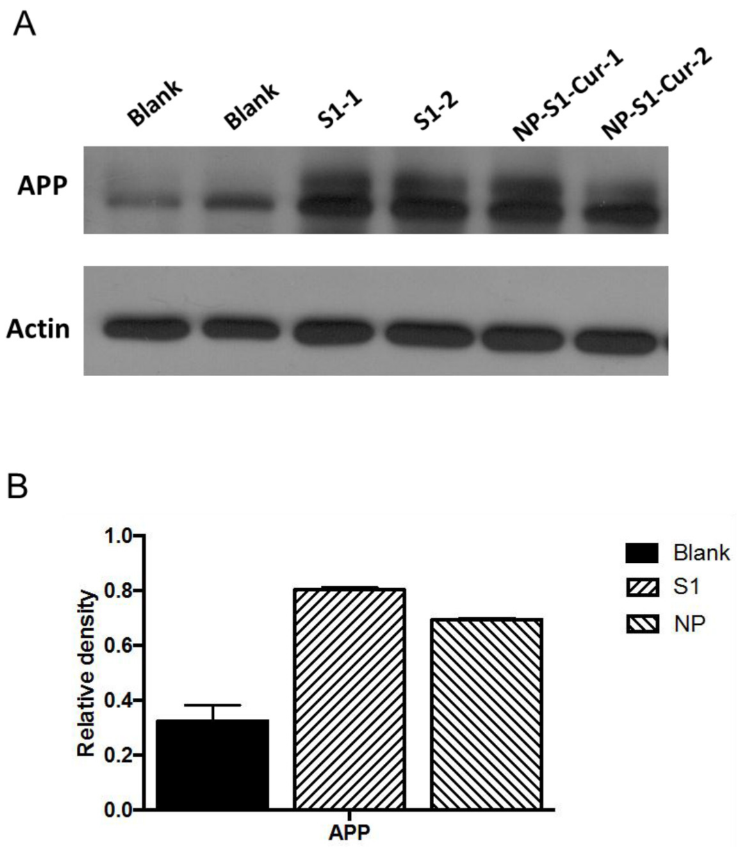

Supplementary Figure 3: S1 peptide and S1 loaded NPs reduced APP cleavage. (A) NP, S1 peptide and NP-S1-Cur was added to 7PA2 cell culture. Cells were collected APP level in cell culture media were analyzed by western blot using the anti-C-terminal of the APP antibody. Actin was used as a loading control. **(B)** Quantification of the western blot using ImageQuant Software.
